# Synergistic Anticancer Efficacy of Curcumin and Doxorubicin Combination Treatment Inducing S-phase Cell Cycle Arrest in Triple-Negative Breast Cancer Cells: An In Vitro Study

**DOI:** 10.7759/cureus.75047

**Published:** 2024-12-03

**Authors:** Esha Sarkar, Afreen Khan, Rumana Ahmad, Aparna Misra, Syed Tasleem Raza, Abbas A Mahdi

**Affiliations:** 1 Department of Biochemistry, Era's Lucknow Medical College and Hospital, Era University, Lucknow, IND

**Keywords:** apoptosis, brca 1 gene, breast cancer therapy, cell cycle arrest, curcumin, dna condensation, p53 gene expression, phytomedicine

## Abstract

Background: Curcumin (Cur) is a polyphenol phyto-compound found in turmeric (*Curcuma longa*) that inhibits tumorigenesis by introducing apoptosis and restricting cell survival and proliferation. This in vitro research article focuses on the pharmacodynamic interactions of Cur combined with the commercial drug doxorubicin (Doxo) to enhance the cytotoxicity of Doxo at lower doses against triple-negative breast cancer cells (MDA-MB-231) with the chemo-protective effect against normal HEK293 cells. In this study, we observed the dose-dependent cytotoxicity, increased reactive oxygen species (ROS) generation, and increased chromatin condensation in combination doses compared to single doses. Moreover, the cell cycle arrest and overexpression of checkpoint regulatory genes *ATM, P53, CHEK2, BRCA-1, *and* BRCA-2* were observed to prevent cell proliferation.

Materials and methods: 3-(4,4-Dimethylthiazol-2-yl)-2,5-diphenyl-2H-tetrazolium bromide (MTT) analysis is performed to determine cell viability at different doses. ROS generation is observed using DCFH-DA-stained fluorescence images. Hoechst33342-stained photomicrographs detect DNA condensation. Apoptosis analysis is performed using Annexin V/FITC and PI flow cytometry. To validate the findings, mRNA expression of cell-cycle checkpoint markers is quantified using reverse transcription quantitative polymerase chain reaction analysis.

Results: The calculated combination dose showing maximum growth inhibition is 33.12 µM Cur + 0.33 µM Doxo against MDA-MB-231 cells with negligible cytotoxicity against normal HEK293 cells. There is a significant increase in mRNA expressions of *P53* (4.43-fold)*, CHEK2* (2.58-fold),*BRCA-1 *(2.01-fold)*, BRCA-2 *(1.60-fold),and* ATM *(0.91-fold) genes (2^-^^∆∆^^Ct^) after treatment with the combination doses, evident with the major S-phase cell cycle arrest in MDA-MB-231 cells.

Conclusion: Cur synergistically chemo-sensitizes the anticancer activity of Doxo and enhances the responses toward conventional chemotherapy attenuating breast cancer.

## Introduction

Breast cancer is the most prevalent cancer among women and the second most common cancer worldwide, affecting approximately 2.3 million women annually. According to WHO's statistical analysis in 2022, 670,000 women were expected to die from breast cancer [1]. Additionally, the most recent report by the American Cancer Society indicated that 45% of newly diagnosed breast cancer cases (820,000) in the United States were projected for the year 2023 [2].

Breast cancer cells can be characterized based on the presence and absence of various receptors like estrogen receptor (ER), progesterone receptor (PR), epidermal growth receptor (HER2), etc. This study is based on the highly metastatic triple-negative breast cancer cell lines, MDA-MB-231 (ER-, PR-, and HER2-), isolated from basal-type carcinoma of the mammary gland [3]. These cells are extremely aggressive, metastatic, and prone to relapse with a poor response toward hormone and drug therapies (doxorubicin (Doxo), paclitaxel, docetaxel, tamoxifen, etc.) due to multidrug drug resistance (MRD) [4].

Doxo, extracted from *Streptomyces peucetius*, is an antibiotic (anthracycline), successfully used as a chemotherapeutic drug to treat breast cancers, along with lung and ovarian cancers, by targeting topoisomerase-II [5]. Despite great evolution in medicinal research, conventional methods are still not effective in breast cancer because of high systemic toxicity, poor pharmacokinetics, and MDR [6]. The serious drawback of prolonged use is acute toxicity to healthy tissues and high cardiotoxicity over time [7]. To combat MRD, a combination of multiple drug regimens (including anthracyclines, taxanes, methotrexate, cyclophosphamide, and fluorouracil) is aimed at inducing cell death more efficiently, even at lower concentrations. Any compound that increases the sensitivity of conventional therapy toward cancer cells and decreases tumor cell survivability in comparatively lower concentrations is called a chemosensitizer [8].

In the search for chemosensitizers, phyto-compounds became the mainstay of recent research in cancer therapeutics, which are present in different parts of plants (fruits, roots, stems, bark, and leaves) and have shown their medicinal properties against various pathological comorbidities, such as allergy, inflammation, diabetes, obesity, hypertension, cardiovascular disease (CVD), various immune and inflammatory diseases, neurological disorders, and cancer [9]. Curcumin (Cur), a polyphenol considered the most abundant phyto-compounds (almost 2-6%) among all 60 different active curcuminoids and non-curcuminoids of *Curcuma longa *or turmeric, has drawn the researcher’s interest due to its wide range of anti-oxidant, anti-inflammatory, and anti-allergic properties [10]. Various studies have reported that Cur acts as an anticancerous compound against breast cancers to induce cell cycle arrest at various phases by decreasing *CDC25 *and *CDC2* and increasing the expression of the *P21* gene [11].

The present study focuses on Cur chemosensitizing apoptosis in the MDA-MB-231 cell line in combination with Doxo. The chemoprotective activity against a normal embryonic kidney HEK293 cell line was also established. However, the underlying mechanisms of programmed cell death were further analyzed with differentially expressed genes involved in cell cycle propagation. The study specifically intends to establish Cur as a complementary medicine in combination with Doxo.

## Materials and methods

Chemical and reagents

DMEM/F-12 (Dulbecco’s Modified Eagle Medium/Nutrient Mixture F-12 Ham) growth media and streptomycin antibiotic/antimycotic solution were purchased from Thermo Fisher Scientific, MA, USA. Cur (C1386), Doxo-HCl (D1515) active compounds, FBS (HiMedia-RM), DCFH-DA (Cat: D6883), and Hoechst33342 (Cat: B2261) were purchased from Sigma-Aldrich, Sigma-Life, MO, USA; MTT dye from HiMedia, India; and Annexin V/FITC-PI apoptosis kit (Cat: K101-100) and PI (K101-100-3) from BioVision, CA, USA. Total mRNA extraction PureLink RNA mini kit (Cat: 12183018A) was obtained from Invitrogen, MA, USA, and cDNA synthesis kit (Cat: 4368814) from Applied Biosciences, MA, USA. For gene expression assay, TaqMan Fast Advanced Master Mix (Cat: 4444556) and TaqMan gene expression assays (for genes *ATM, P53, CHEK2, BRCA-1, BRCA-2, *and *GAPDH*) were obtained from Thermo Fisher Scientific, MA, USA.

Culture and maintenance of cell lines

MDA-MB-231 and HEK293 cells were obtained from the NCCS, Pune, India, and maintained by sub-culturing and passaging as monolayers in cell culture flasks (Nest; Tarsons, India) at 37°C in a 5% CO2 incubator at 95% humidity (Tissue and Cell Culture Laboratory, Era's Medical College, Era University, Lucknow, India). The cells were cultured in DMEM/F-12 and added with 10% FBS and 1% streptomycin.

Preparation of drug combination

Powdered Cur (C1386) and Doxo-HCl (D1515) were dissolved in 0.5% DMSO and stored at 4°C. The combination of Cur and Doxo was made using Chou-Talalay’s combination index (CI) method to quantify synergism, additive, and antagonist effects. The CI isobologram equation allows quantitative determination of drug interactions based on the median-effect equation, where CI<1, CI=1, and CI>1 indicate synergism, additive effect, and antagonism, respectively. CompuSyn (Biosoft, Cambridge, UK), a computerized programming software based on these techniques, is used to automatically determine the synergism or antagonism of multiple drugs at any effective dose [12].

Cytotoxicity and cell viability detection using MTT assay

The cytotoxicity and anti-proliferative activities of Cur, Doxo, and their combination doses against MDA-MB-231 and HEK293 cells were measured using the 3-(4,4-dimethylthiazol-2-yl)-2,5-diphenyl-2H-tetrazolium bromide (MTT) assay; 1 × 104 cells/mL cells were seeded in 96-well culture plates and incubated overnight at 70% confluency for drug treatment. Various concentrations of Cur (5-95 μM) and Doxo (0.25-5 μM) were implemented to acquire dose- and time-dependent (24, 48, and 73 hours) cell viability analysis. Based on the IC50 concentrations, the Cur and Doxo combination dose was prepared using the CompuSyn software. To calculate cell viability, MTT dye (5 mg MTT/1 ml PBS) was applied, which forms purple formazan reduced by mitochondrial oxidoreductases. An ELISA reader (800TS microplate reader, BioTek, VT, USA) was used to obtain the OD at dual filters (OD_595_/OD_630_). The %Cell viability of each dose was calculated compared with the control (formula), and the graph was generated using GraphPad Prism 8 (Insight Venture Management, LLC, NY, USA).

% Cell-viability: (OD of Test _average_/OD of Control _average_) ​​​​​x 100 ​(formula)

The cell morphologies were observed using a phase-contrast microscope (Nikon, Shinagawa, Japan), and micrographs were collected using NIS-Element software (Nikon Instruments, Tokyo, Japan).

Measurement of oxidative stress (ROS generation)

Intracellular reactive oxygen species (ROS) generation was measured using DCFH-DA staining (5-(-6)-carboxy-2,7-dichlorofluorescein diacetate). After 48 hours of treatment, the control and treated cells were incubated with DCFH-DA (10 μM in 1X PBS) for 20 minutes at room temperature (RT: 20-25°C). Fluorescence was captured using a phase-contrast microscope at 485 nm (Zeiss AxioVert, Oberkochen, Germany).

Analysis of dsDNA condensation

The nuclear dsDNA condensation was measured using the Hoechst33342 (Cat: B2261) stain, which binds at the AT-rich sequence of the minor groove and is usually utilized to determine cell cycle status and apoptosis stage. Cells were fixed with methanol and glacial acetic acid (3:1) and incubated with Hoechst (2 μg/ml PBS) at room temperature for 20 minutes at RT (dark) [13]. The nuclear morphology and stages of condensation were observed under an inverted fluorescence microscope.

Determination of apoptosis stages

The percentage of viable, apoptosis (early and late), and dead cells at different drug doses was quantified using a flow cytometer by Annexin V-FITC and PI Apoptosis Kit (Cat: K101-100) following the user’s guidelines. Forty-eight hours after treatment, the treated cells were stained with Annexin V-FITC (2μl) and PI (2μl) for 15 minutes at RT (dark). The apoptosis index was analyzed using a flow cytometer (FACSCanto II Clinical Flow Cytometry System, BD Biosciences, CA, USA) [14].

Analysis of cell cycle stages

Cell cycle arrests at different stages were analyzed by calculating the ratio of cells in the G_0_/G_1_, S, and G_2_/M phases of the cycle. After 48 hours of treatment, MDA-MB-231 cells were fixed using 70% cold ethanol (-20°C for two hours), followed by permeabilization with 0.2% triton (37°C for 30 minutes), and incubation with RNase-A (Sigma) for 30 minutes. Stained with 10 µl PI (K101-100-3), the cells were analyzed using a flow cytometer.

Expression of regulatory marker genes using RT-qPCR method

The genetic expressions of apoptotic markers were analyzed using the RT-qPCR method. Total cellular RNA was extracted and purified using the PureLink RNA Mini Kit (Cat: 12183018A). Eluted RNA was re-suspended in RNase-free water (Ambion, USA) and quantified with a NanoDrop 2000 spectrophotometer (Thermo Fisher Scientific, MA, USA). First-strand cDNA was synthesized using the High-Capacity cDNA Reverse Transcription Kit (Cat: 4368814) following the PCR amplification: step 1: 25°C for 10 minutes; step 2: 37°C for 120 minutes; step 3: 85°C for five minutes; and step 4: 4°C for ∞ time [14]. RT-qPCR analysis was performed using the TaqMan Fast Advanced Master Mix (Cat: 4444556) and TaqMan gene expression assays (Table 1) in an RT-PCR machine (Applied Biosystems, StepOnePlus system version 2.3, Canada). The expression analysis follows the steps of denaturation (95°C: 21 seconds) and annealing with the primers of the TaqMan gene expression assay (60°C: 20 seconds). The relative expression of *BRCA-1, BRCA-2, P53, ATM,* and *CHEK2* genes was calculated with comparative fold change (2^-∆∆Ct^) values, where *GAPDH* was used as an endogenous control.

**Table 1:** TaqMan gene expression assay IDs

Target gene	Chromosome	Amplicon length	Assay ID	Cat. No.	Dye	Annealing temp.
ATM	Chr.11q22-23	89	Hs00175892_m1	4453320	FAM^TM^	60°C
P53	Chr.17p13.1	108	Hs01034249_m1	4453320	FAM^TM^	60°C
CHEK2	Chr.22q12.1	109	Hs00200485_m1	4453320	FAM^TM^	60°C
BRCA-1	Chr.17q12.12	59	Hs01556193_m1	4453320	FAM^TM^	60°C
BRCA-2	Chr.13q12.3	110	Hs00609073_m1	4453320	FAM^TM^	60°C
GAPDH	Chr.12p13.31	157	Hs02786624_g1	4453320	FAM^TM^	60°C

Statistical analysis

Cell viability and mean fluorescence intensity data were expressed as mean ± SD or mean ± SEM from three independent experiments. Statistical evaluation would be determined by one-way or two-way ANOVA followed by Dunnett’s multiple comparison test using GraphPad Prism 8 software. A p-value <0.05 would be considered statistically significant.

## Results

Synergism of the Cur and Doxo combination treatment on cell morphology and viability

**Figure 1:**
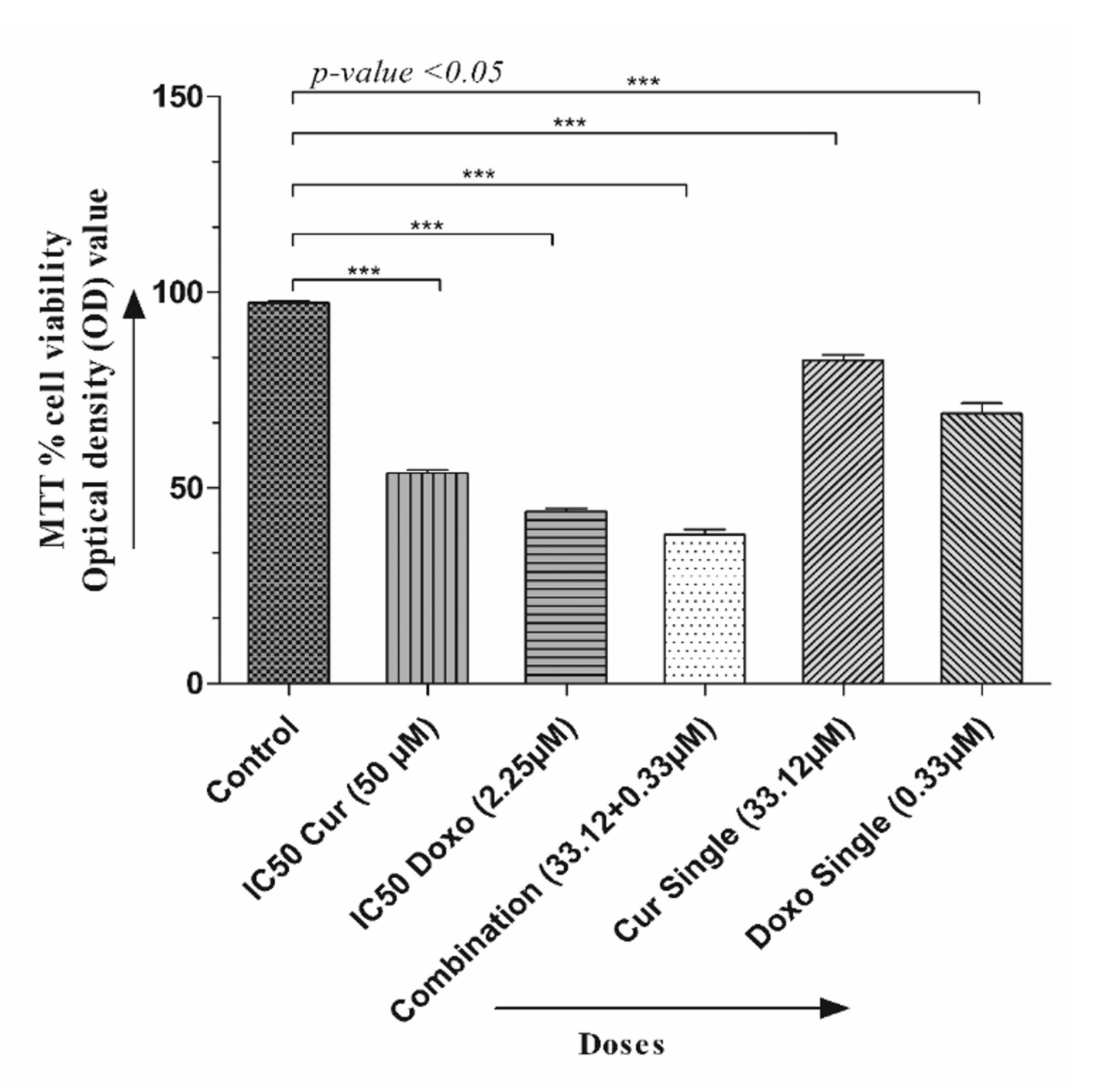
Graphical presentation showing the MTT cell viability assessment of MDA-MB-231 cells with different drug doses (48 hours). Each column presents the mean ± SEM of triplicates compared with the control; p<0.05 is statistically significant MTT: 3-(4,4-dimethylthiazol-2-yl)-2,5-diphenyl-2H-tetrazolium bromide, SEM: standard error of the mean, Cur: curcumin, Doxo: doxorubicin

**Figure 2:**
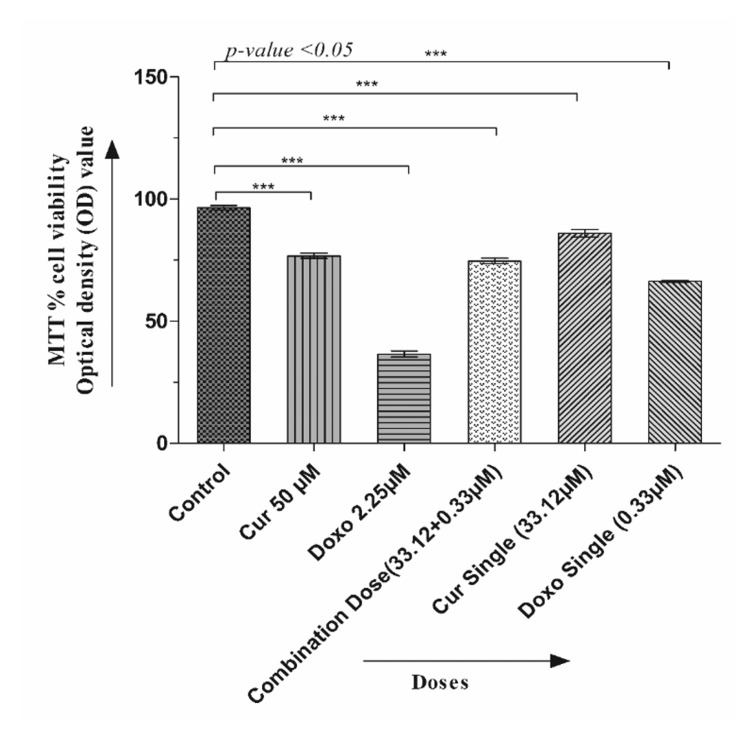
Graphical presentation showing the chemo-protective efficacy of Cur on normal HEK293 cells in the Cur and Doxo combination treatment (48 hours). Each column presents the mean ± SEM of triplicates compared with the control; p<0.05 is statistically significant MTT: 3-(4,4-dimethylthiazol-2-yl)-2,5-diphenyl-2H-tetrazolium bromide, SEM: standard error of the mean, Cur: curcumin, Doxo: doxorubicin

**Figure 3:**
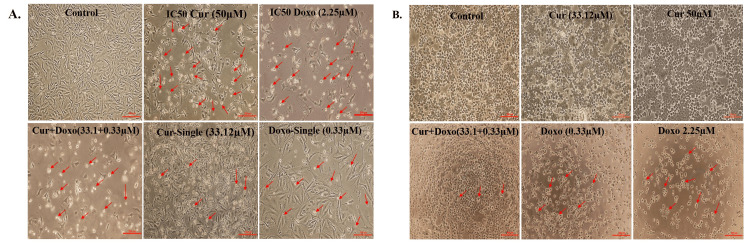
A: Micrographs showing the synergistic cytotoxicity of the Cur and Doxo combination treatment (33.12 µM Cur + 0.33 µM Doxo) against MDA-MB-231 cells (48 hours). B: Micrographs showing the chemo-protectiveness of Cur in the Cur and Doxo combination treatment against HEK293 cells (48 hours). The red arrow shows the dead or apoptotic cells; scale bar = 200 µm Cur: curcumin, Doxo: doxorubicin

The dose-effective growth curves were analyzed after treatment with Cur, Doxo, and their combinations. The MMT analysis showed a decrease in cell viability in a time-based manner (24, 48, and 72 hours), among which 48-hour drug treatment was selected for detecting IC50 (50% viability) and further staining analysis. Cur and Doxo showed IC50 at 50 μM and 2.25 μM concentrations. The morphological changes after the 48-hour combination treatment were demonstrated in Figure 1A, where untreated cells showed the usual adherence and surface characteristics. The maximum number of non-adherent and floating spherical MDA-MB-231 cells were visible after the combination treatment (33.12 μM Cur + 0.33 μM Doxo), indicating apoptosis. The synergistic effect was predicted by CompuSyn using the dose (μM) and inhibitory effects (%) of the two drugs (Table 2, Figure 2). Meanwhile, the combination drug-treated HEK293 cells showed no significant alteration in cell morphology (Figure 1B) and viability, proving Cur's chemo-protectiveness (Figure 3).

**Table 2:** Dose-dependent cytotoxicity assessment of Cur and Doxo and synergistic effects of the Cur and Doxo combination treatment on MDA-MB-231 cells (analyzed using the CompuSyn software) Dm: The ME dose, in this case, is the IC50 value, which indicates “potency.” The value can be obtained from the X-intercept of the ME plot. Dm1: IC50 of Cur, Dm2: IC50 of Doxo-HCl, Dm3: IC50 of the combined dose. m: slope (signifies the shape of the curve in the ME plot, m=1, >1, and <1), m1: the slope of Cur, m2: the slope of Doxo-HCl, m3: the slope of the combined dose. r: The linear correlation coefficient of the ME plot. It signifies the “conformity” of the data with the mass-action law, an indication of how good are the data, when r=1, it is perfect. For in vitro experiments, usually r>0.95 is considered good, r1: correlation coefficient of Cu, r2: correlation coefficient of Doxo-HCl, r3: correlation coefficient of the combined dose. CI: combination index (CI<1: synergism, CI=1: additive, and CI>1: antagonist) Cur: curcumin, Doxo: doxorubicin, ME: median effect

D1: Cur (μM)	Cur toxicity	m1	Dm1 (µM)	r1	CI	DRI (Cur)	Conclusion
10.0	0.05	1.31 ± 0.38	42.60 µM	0.65	NIL	NIL	Dose-dependent cytotoxicity of Cur
15.0	0.09						
20.0	0.15						
25.0	0.21						
30.0	0.27						
35.0	0.32						
40.0	0.35						
45.0	0.43						
50.0	0.50						
55.0	0.54						
60.0	0.58						
65.0	0.62						
70.0	0.65						
75.0	0.69						
80.0	0.72						
85.0	0.76						
90.0	0.81						
95.0	0.85						
D2: Dox (μM)	Doxo toxicity	m2	Dm2 (µM)	r2	CI	DRI (Dox)	Conclusion
0.25	0.14	1.3 ± 0.21	1.74 µM	0.89	NIL	NIL	Dose-dependent cytotoxicity of Doxo-HCl
0.5	0.18						
1.0	0.25						
1.25	0.31						
1.5	0.35						
2.0	0.40						
2.25	0.49						
2.5	0.53						
3.0	0.68						
3.25	0.74						
3.50 4.0	0.78						
	0.91						
D1 + D2: Cur + Doxo (μM in 100:1)	Simulated toxicity	m3	Dm3 (µM)	r3	CI	DRI (Cur + Doxo)	Conclusion
25.0 + 0.25	0.37	2.12 ± 0.26	33.4478 µM	0.95	1.10	1.13: 4.61	Synergism with favorable dose reduction
30.0 + 0.30	0.49				0.91	1.38: 5.61	
33.12 + 0.33	0.50				0.90	1.29: 5.24	
35.0 + 0.35	0.54				0.91	1.38: 5.61	
40.0 + 0.40	0.58				0.91	1.36: 5.57	
50.0 + 0.50	0.62				1.00	1.24: 5.06	
55.0 + 0.55	0.71				0.81	1.54: 6.29	
60.0 + 0.60	0.75				0.76	1.65: 6.74	
65.0 + 0.65	0.81				0.63	1.988: 8.155	
70.0 + 0.70	0.89				0.412	3.015: 12.399	

Cur and Doxo induce ROS generation in combination treatment

**Figure 4:**
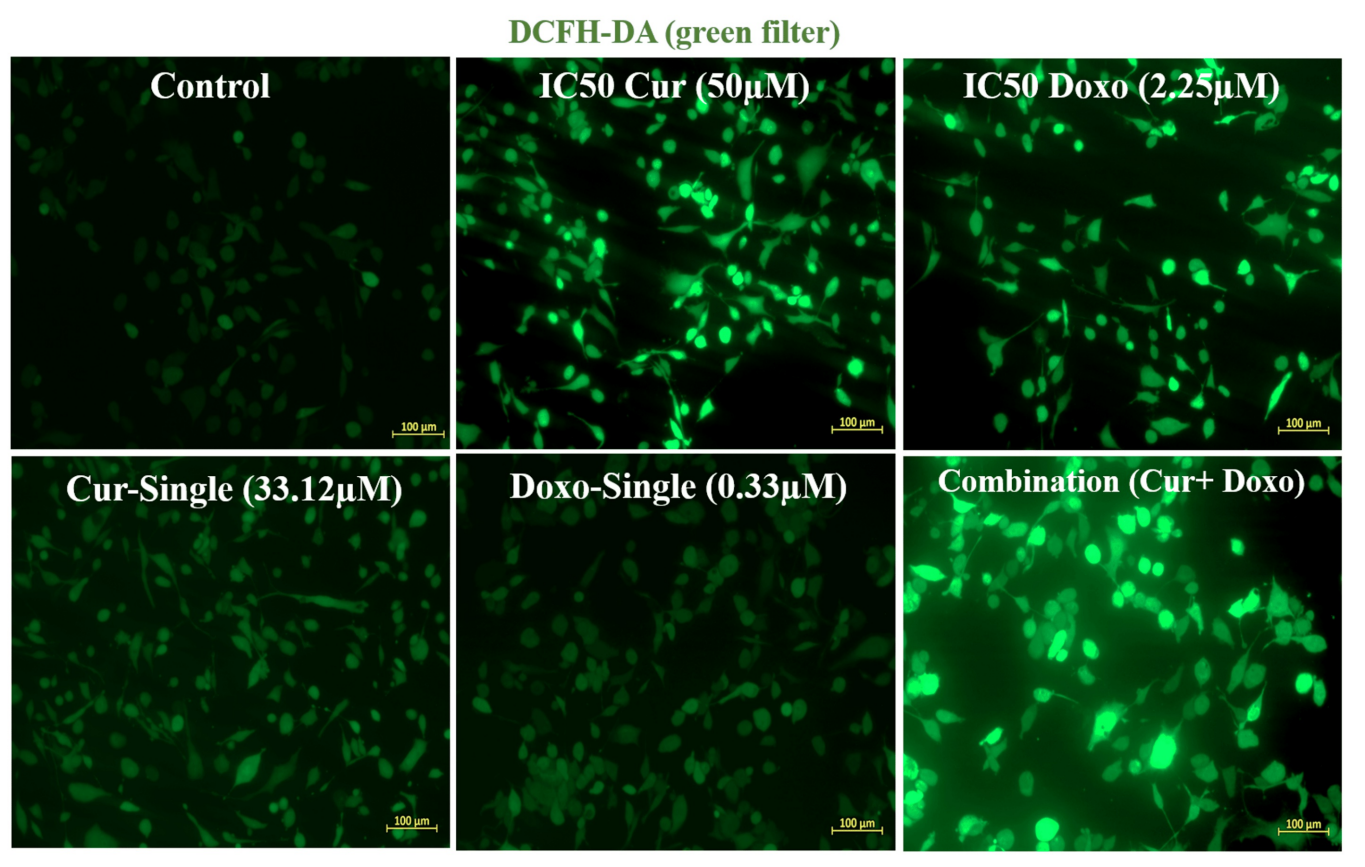
Cur- and Doxo-induced elevation of intracellular ROS level in MDA-MB-231 cells. Fluorescent images show highly elevated intracellular ROS in Cur and Doxo combination dose-treated MDA-MB-231 cells (48 hours) than other doses Cur: curcumin, Doxo: doxorubicin, ROS: reactive oxygen species

**Figure 5:**
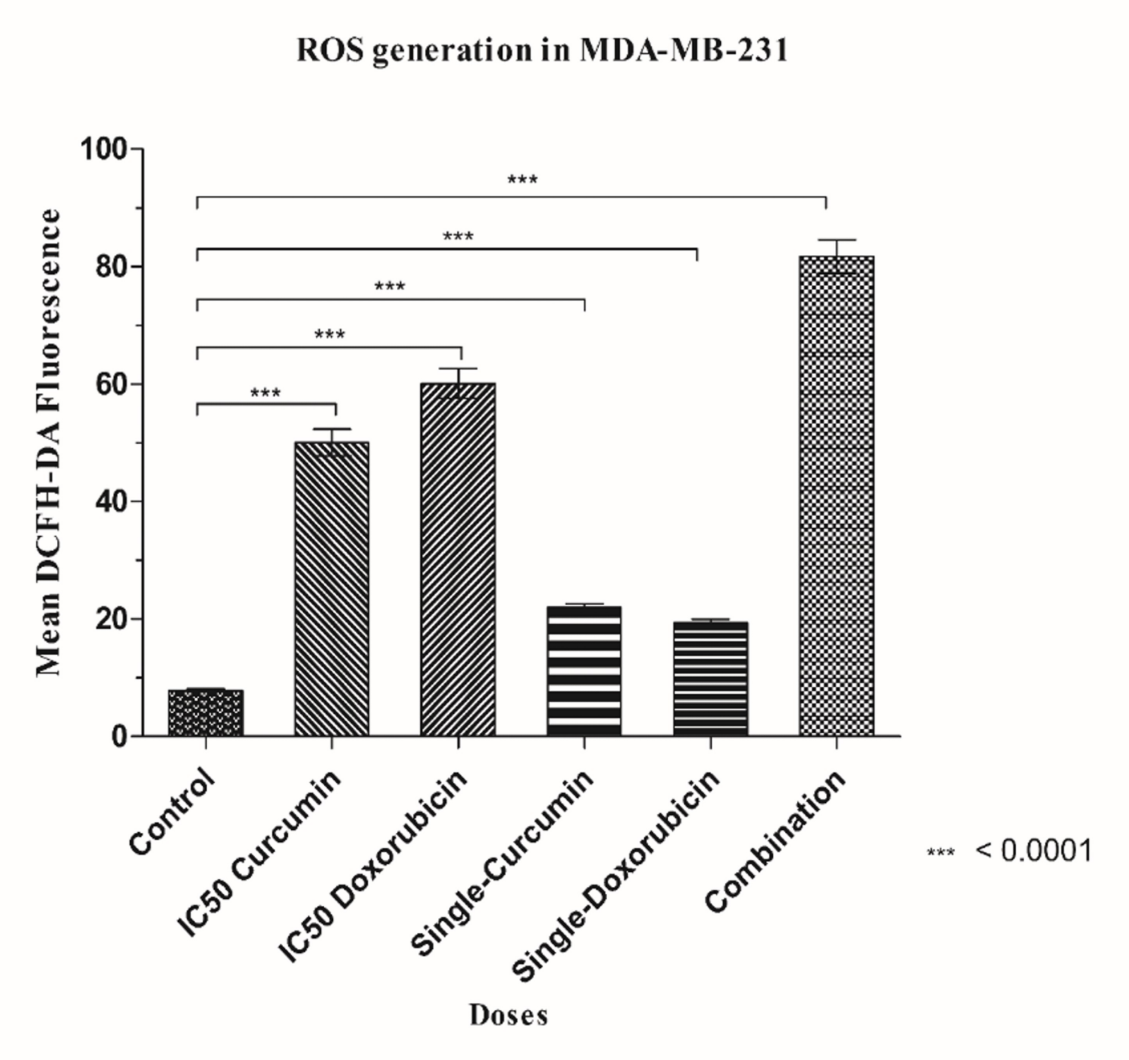
Graphical analysis of mean DCFH-DA fluorescence in different dose-treated MDA-MB-231 cells. Column values were analyzed as mean ± SD of triplicates; p<0.05 is statistically significant ROS: reactive oxygen species, SD: standard deviation

Figure 4 depicts that the cellular oxidative stress increased significantly in treated cells compared to untreated cells. The elevated ROS fluorescence intensity was quantified using ImageJ software (National Institutes of Health, MD, USA). Due to synergy, the combination drug-treated cells resulted in the highest ROS generation than the IC50 concentrations of the drugs when taken separately (Figure 5). This result suggests that a probable mechanism for the onset of early apoptosis might be elevated oxidative stress.

Induced chromatin condensation after Cur and Doxo combination treatment

**Figure 6:**
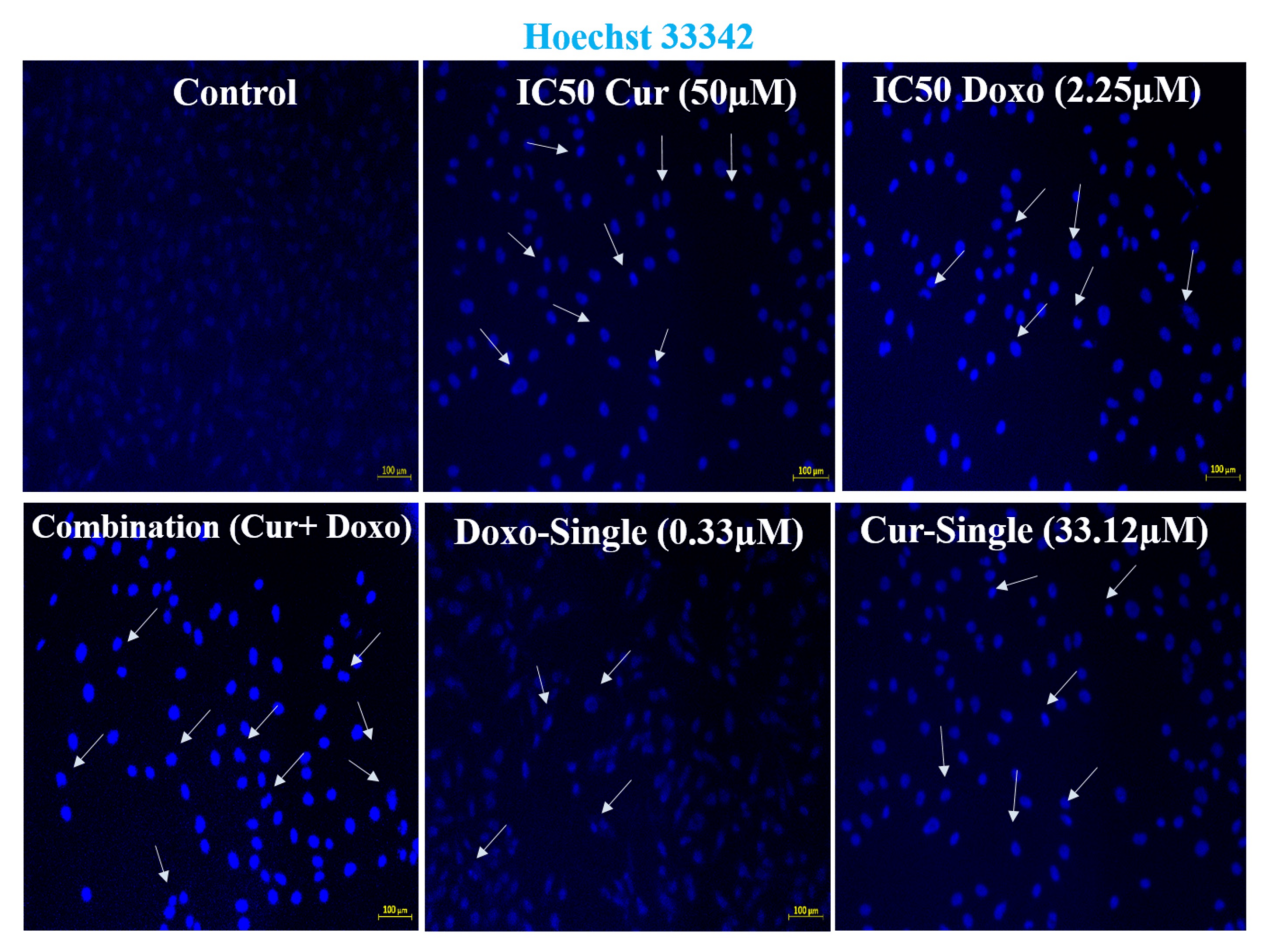
Hoechst33342 dye-stained fluorescent images indicating the drug-induced chromatin condensation and DNA break, where Cur and Doxo combination dose-treated MDA-MB-231 cells showed the highest number of condensed nuclei compared to IC50 and single doses of drugs (treated alone); scale bar = 100 µm DNA: deoxyribonucleic acid, Cur: curcumin, Doxo: doxorubicin

**Figure 7:**
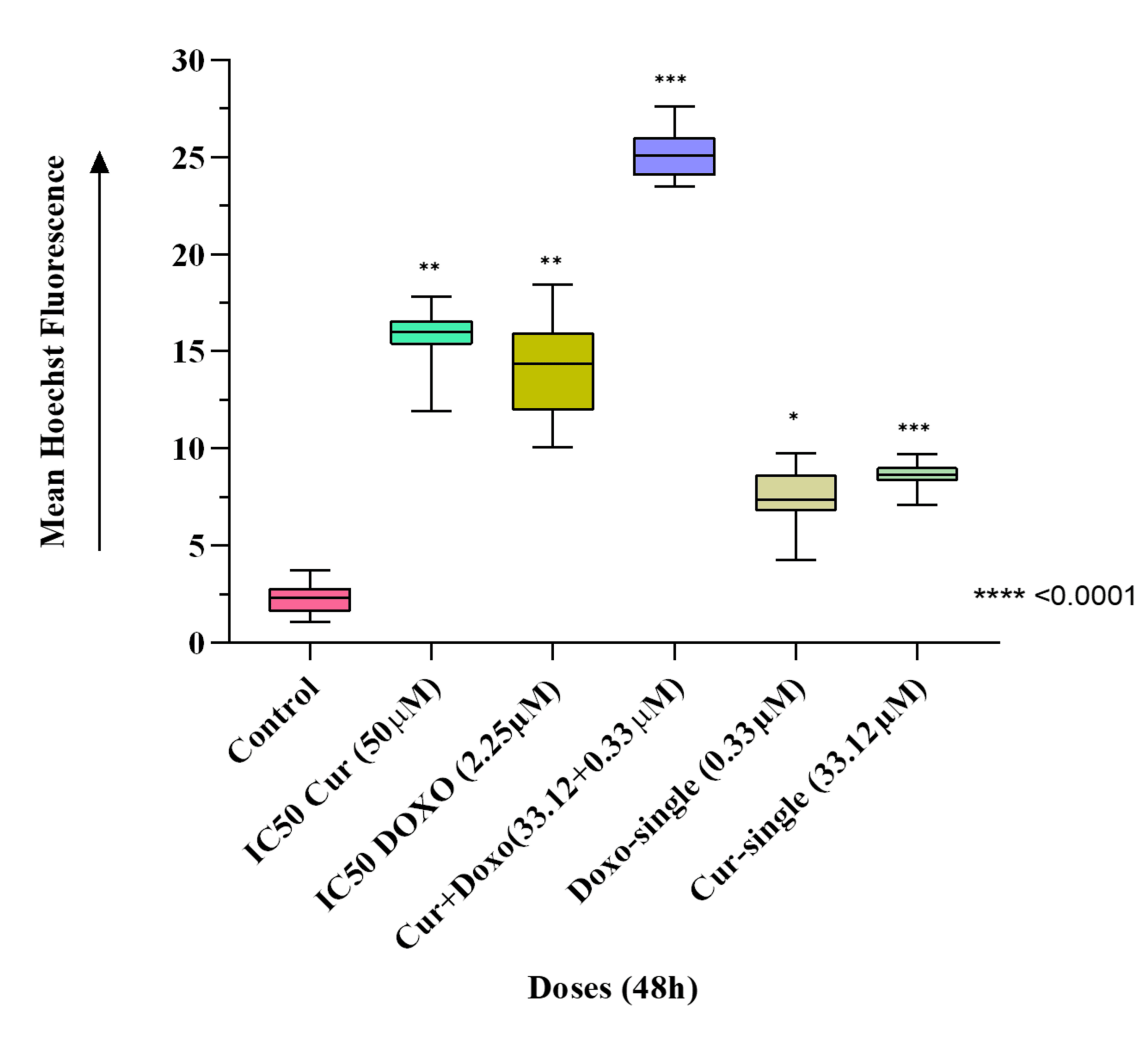
Graphical analysis of mean Hoechst33342 fluorescence in different dose-treated MDA-MB-231 cells. Column values were analyzed as mean ± SD of triplicates; p<0.05 is statistically significant SD: standard deviation, Cur: curcumin, Doxo: doxorubicin

The fluorescence microscopic observation (Figure 6) evidences the highly condensed chromatin fiber and apoptotic body formed in the combination dose-treated MDA-MB-231 cells compared to untreated cells. Also, comparatively less chromatin condensation was visible in the single doses and IC50 dose-treated MDA-MB-231 cells (Figure 7). This indicates enhanced nucleosome cleavage and dsDNA break, driven by ATP depletion and elevated ROS generation [15].

Quantification of apoptosis stages in MDA-MB-231 cells

**Figure 8:**
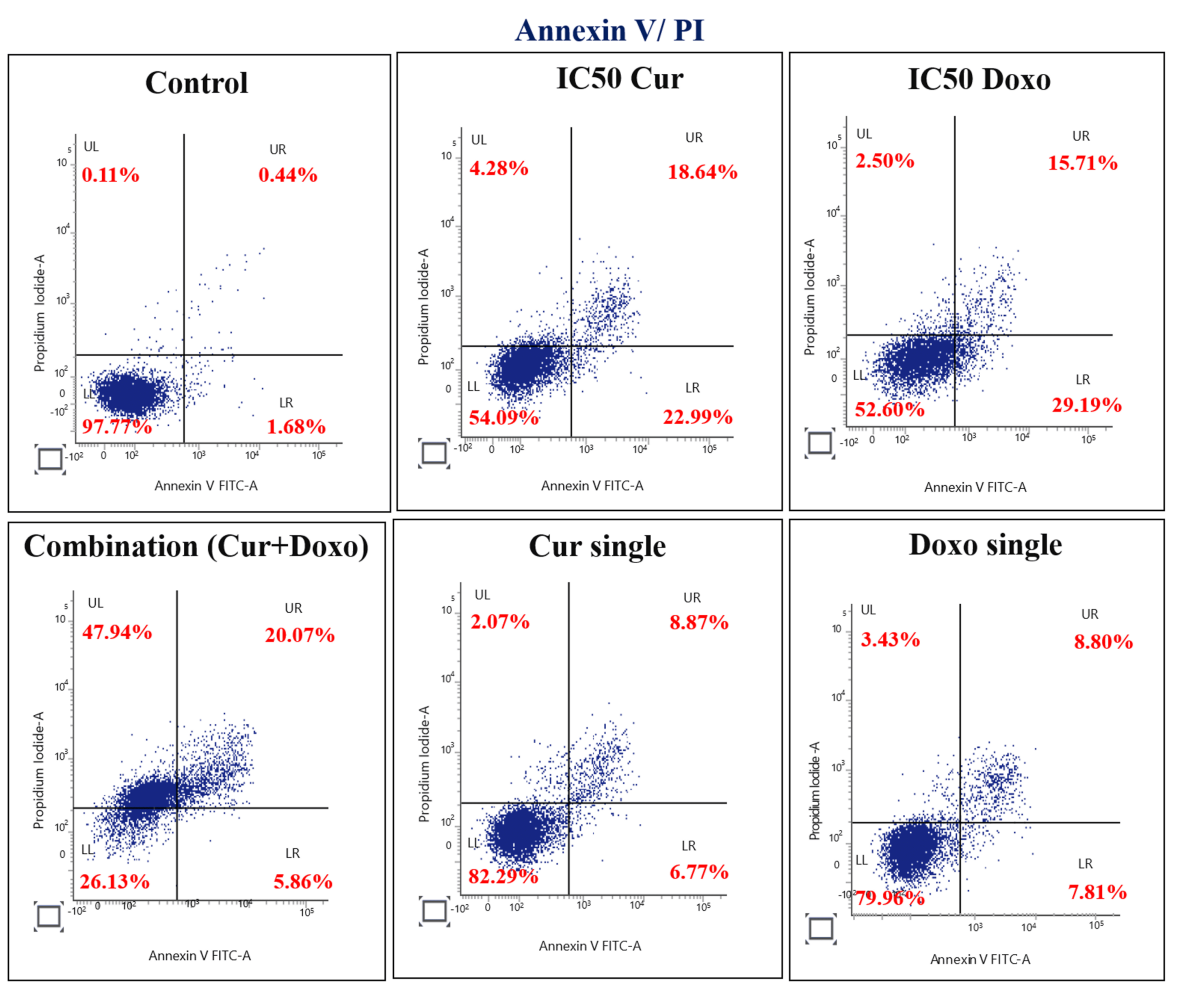
Flow cytometry analysis of apoptosis stages in various dose-treated MDA-MB-231 cells for 48 hours LL: viable cells, LR: early apoptotic cells, UR: late apoptotic cells, UL: dead cell, Cur: curcumin, Doxo: doxorubicin

**Figure 9:**
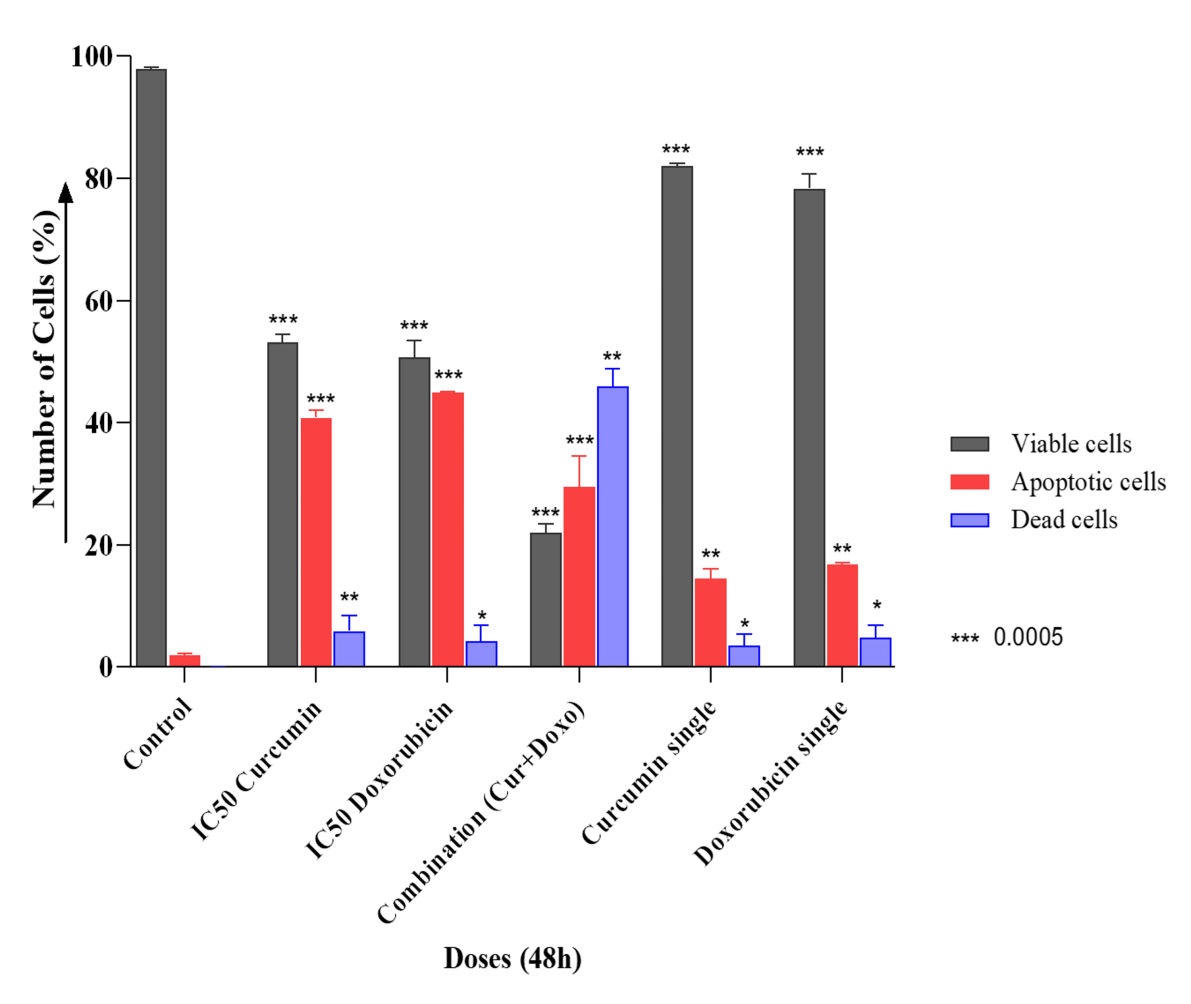
Graphical presentation showing the percentage of viable, apoptotic, and dead cells in different drug doses (48 hours) analyzed using flow cytometry. Bar values were calculated in mean ± SD of duplicates; p<0.05 is significant SD: standard deviation, Cur: curcumin, Doxo: doxorubicin

The apoptotic effect of the Cur and Doxo combination treatment on MDA-MB-231 cells was further quantified by flow cytometry analysis and stained with Annexin V-FITC and the PI double staining method. The results (Figure 8) showed that the untreated sample exhibited 97.77% healthy and viable cells. The IC50 Cur dose-treated MDA-MB-231 cells had 54.09% viability, and the IC50 Doxo treatment exhibited 52.60% healthy cells. A remarkable increase in cellular apoptosis was observed in the combination treatment, where only 26.13% of cells were found live, 5.86% were in early apoptosis, 20.07% were in late apoptosis, and the remaining 47.94% were dead cells. In the single doses of Cur and Doxo, very minimal apoptosis was visible (Figure 9).

Cur and Doxo combination treatment induces S-phase cell-cycle arrest

**Figure 10:**
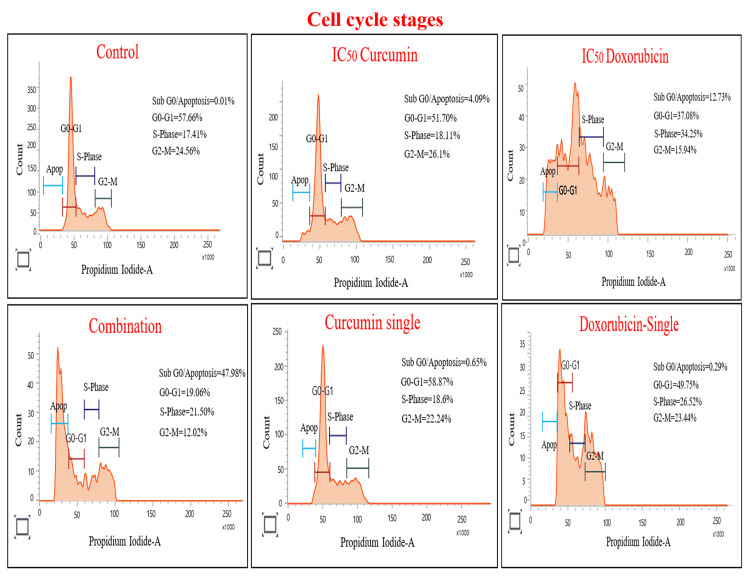
Flow cytometry analysis representing the percentage of cells in different phases of the cell cycle after Cur and Doxo combination treatment on MDA-MB-231 cells (48 hours) indicates the cell cycle arrest Cur: curcumin, Doxo: doxorubicin

**Figure 11:**
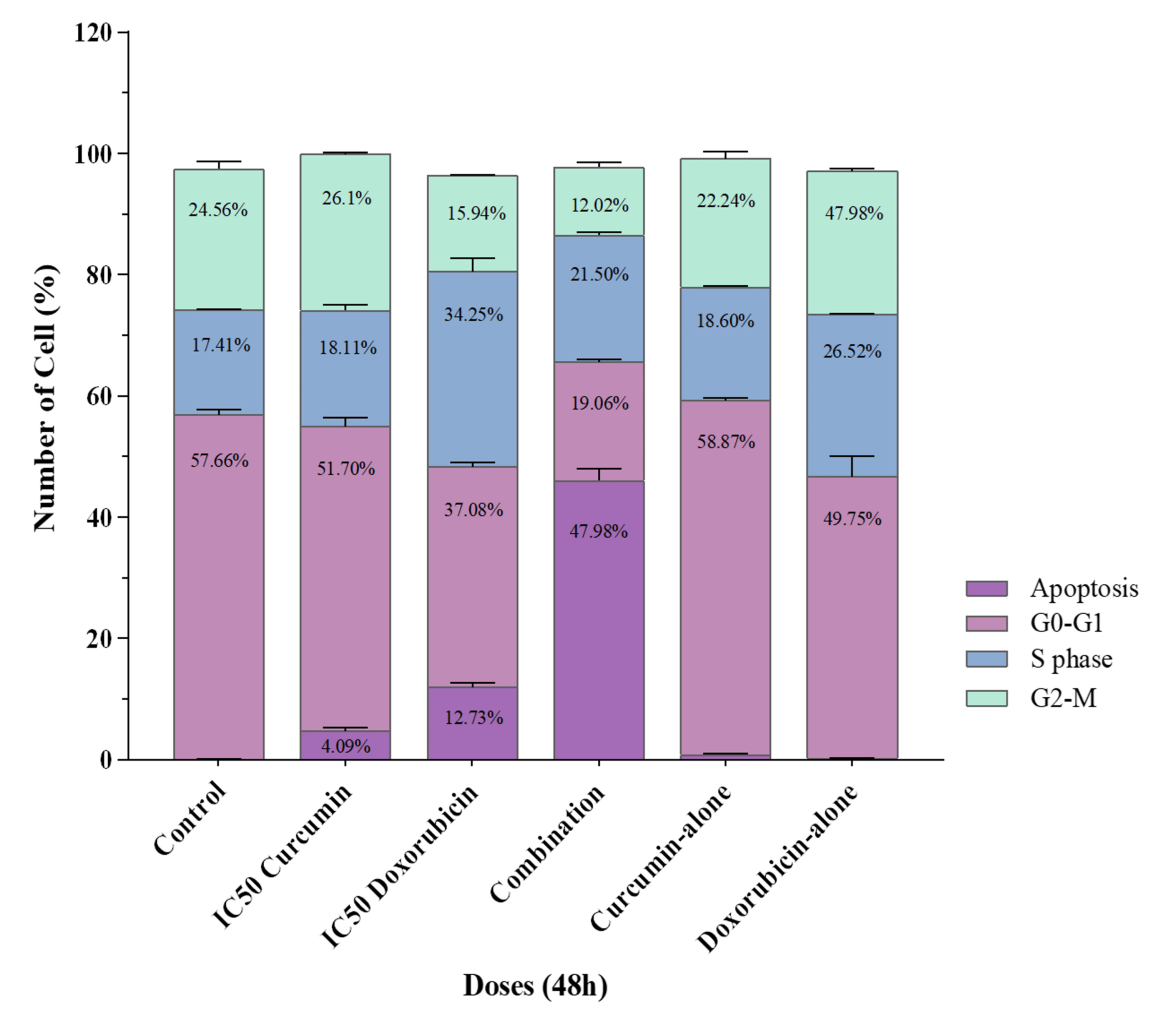
Graphical presentation showing flow cytometry data of cell distribution in different phases of the cell cycle. Bar values were calculated in mean ± SD of duplicates; p<0.05 is significant SD: standard deviation

Figure 10 shows that the IC50 Cur dose increased the cell percentage in the G_2_-M phase (26.1%), while the IC50 Doxo treatment increased the cell count (34.25%) in the S-phase by reducing the cell number in the G0-G_1 _phase. This indicates the G_2_-M phase restriction with Cur and S-phase restriction with Doxo drug treatment. On the other hand, maximum cells were analyzed in the sub G_0_/apoptotic phase (47.99%) with a restriction at the S-phase (21.50%). These results (Figure 11) emphasize the G_1_/S checkpoint activation and S-phase cell-cycle arrest after the Cur and Doxo combination treatment against MDA-MB-231 cells.

Quantitative expression of cell-cycle regulatory genes in treated MDA-MB-231 cells

**Figure 12:**
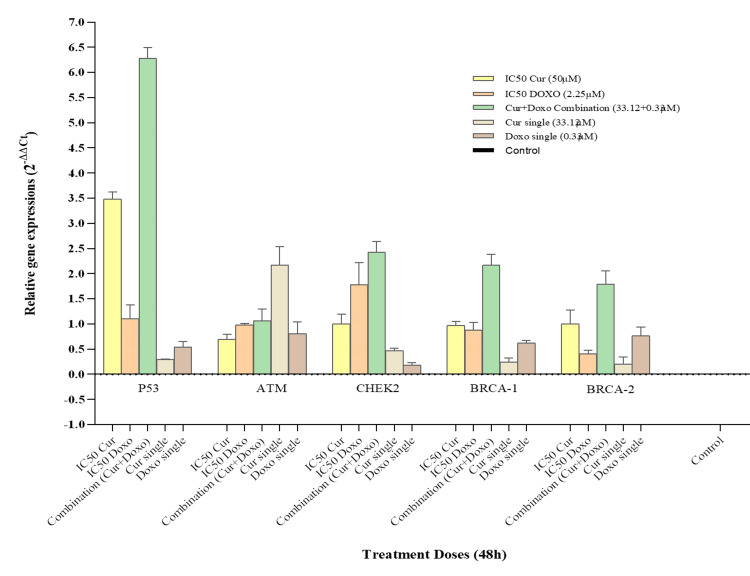
Bar diagram showing relative quantitative expression of cell cycle regulatory genes, ATM, P53, CHEK2, BRCA-1, and BRCA-2 compared between the treated and non-treated MDA-MB-231 cells by RT-qPCR (fold change: 2-∆∆Ct) method. Bar values were calculated in mean ± SD of duplicates; p<0.05 is statistically significant RT-qPCR: reverse transcription quantitative polymerase chain reaction, SD: standard deviation, Cur: curcumin, Doxo: doxorubicin

**Figure 13:**
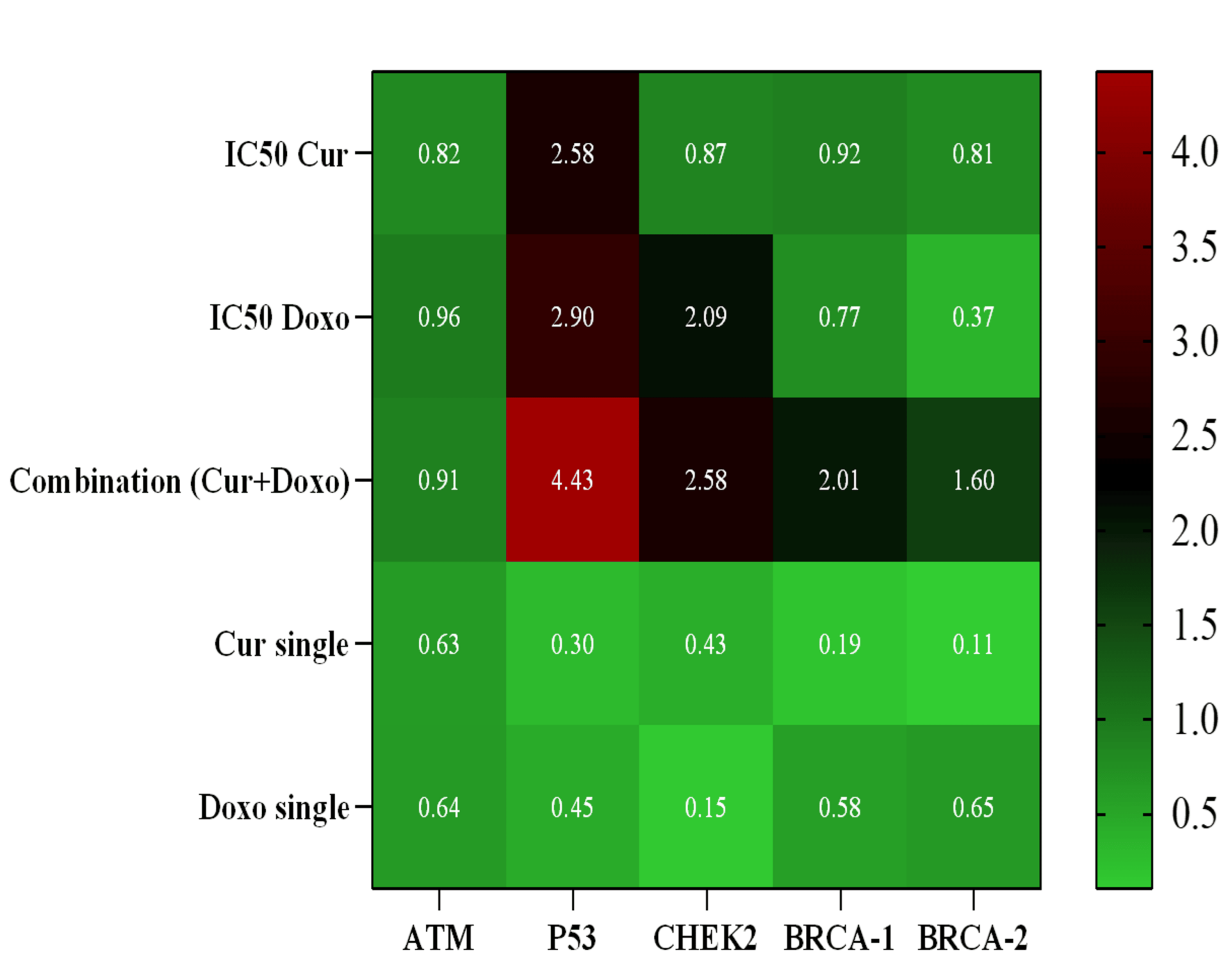
Heat map showing fold change (2-∆∆Ct) of all genes in different dose-treated MDA-MB-231 cells (48 hours) Fold change intensity is presented by color coding, from the lowest fold change +0.15 (green) to the highest fold change +4.43 (red). Cur: curcumin, Doxo: doxorubicin

The quantitative expression of cell cycle regulatory genes (*BRCA-1, BRCA-2, P53, ATM*, and *CHEK2*) was performed with the cDNA of different dose-treated MDA-MB-231 cells. The Cur and Doxo combination dose-treated cells revealed significant upregulation of the *P53* gene (4.43-fold increase) with a moderate elevation of *CHEK2 *(2.58-fold), *BRCA-1* (2.01-fold), and *BRCA-2* (1.60-fold) gene expressions. In the IC50 Cur dose-treated cells, upregulation of the *P53* gene (2.58-fold) was observed, whereas the IC50 Doxo caused an increase in both *P53* (2.90-fold) and *CHEK2* (2.09-fold) gene expressions. However, some minor upregulation of gene expressions was visible in the single doses of Cur and Doxo (Figure 12, Figure 13). The results indicate that *P53* is the gene responsible for restricting the S-phase of the cell cycle in combination with drug treatment.

## Discussion

Among all the breast cancer cells, MDA-MB-231 cells are considered highly relapsing and drug-resistant due to their non-responsiveness toward hormone therapy. Moreover, it also causes numerous adverse effects like skin blisters, hair loss, kidney failure, cardiac toxicity, and multi-organ failure. To deal with these complexities, phyto-extracts or active compounds are widely used with standard therapeutics to minimize or reverse the side effects. Based on the numerous previously reported successful preclinical and clinical trials [16-18], we intended to investigate the cascade mechanism of Cur’s anticancer efficacy. Here, we emphasized increasing the anticancer activities of Doxo combined with Cur at a non-toxic concentration. The MTT assay (Figure 1, Figure 2) observed dose-dependent cytotoxicity with Cur and Doxo treatments when applied alone. However, a synergistic apoptosis rate was evident when applied in combination (33.12 μM Cur + 0.33 μM Doxo) [14]. Further results of elevated oxidative stress (Figure 4, Figure 5) speculated that ROS generation could be a reason behind altered intracellular homeostasis [19], leading to nuclear condensation in apoptotic cells (Figure 6, Figure 7) [20]. The flow cytometry data provided statistical information about the staging of apoptosis (Figure 8) and the cell cycle arrest. The Cur and Doxo combination treatment increased cell number in the S-phase, indicating an S-phase arrest in treated MDA-MB-231 cells (Figure 10).

**Figure 14:**
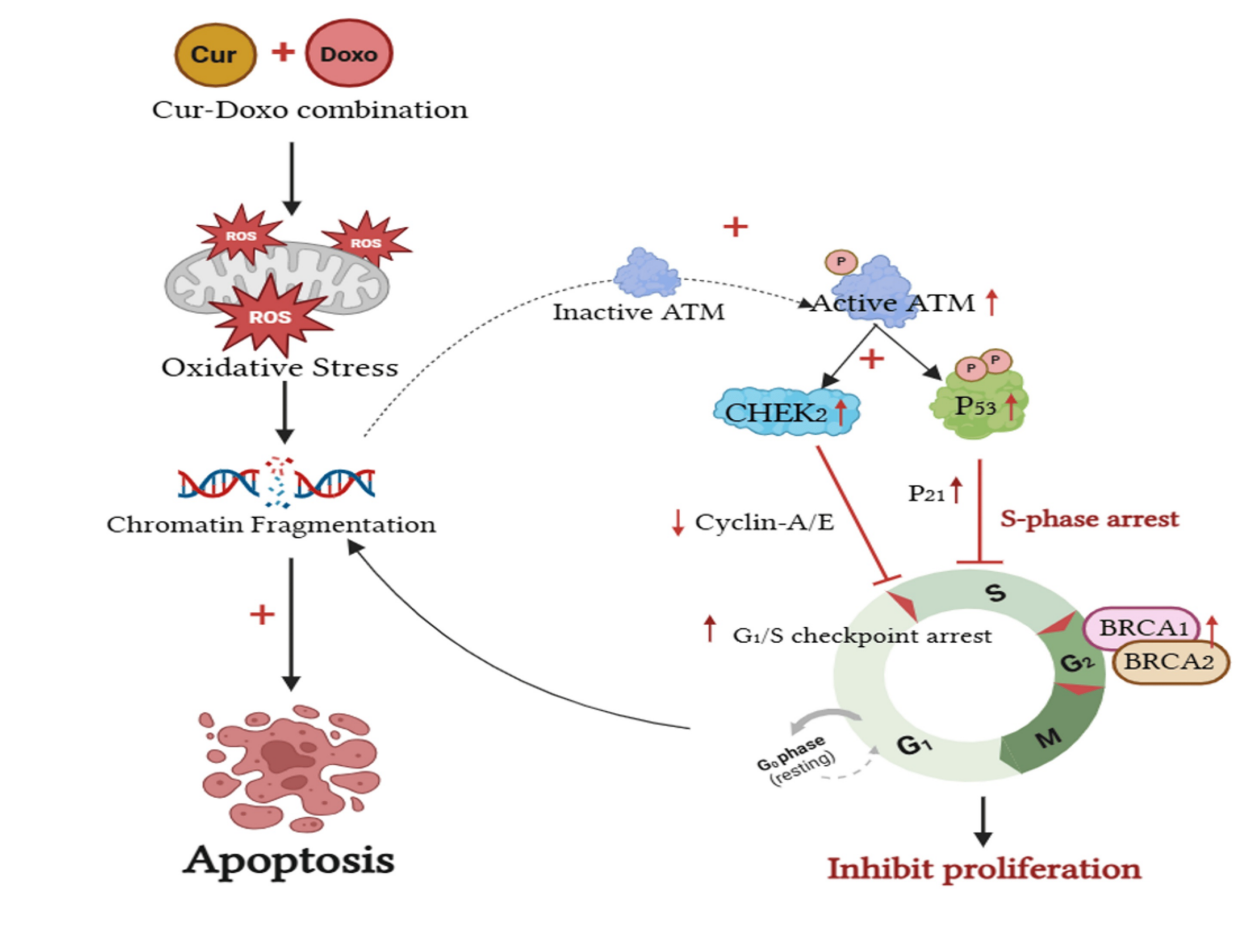
Schematic presentation explaining the underlying mechanism of chemo-sensitizing apoptosis in MDA-MB-231 cells by Cur combined with Doxo. The Cur and Doxor combination treatment enhances the oxidative stress downstream of chromatin condensation and fragmentation. The S-phase cell cycle arrest takes place by significant upregulation of ATM, P53, CHEK2, BRCA-1, and BRCA-2 gene expression Image Credit: Author (generated using www.BioRender.com) Cur: curcumin, Doxo: doxorubicin

Moreover, the gene expression results revealed the significant elevation of the tumor suppressor *P53* gene expression by 4.43-fold (role in restricting S-phase) along with the upregulation of G_1_-S checkpoint regulator *CHEK2* (2.58-fold), *BRCA-1* (2.01-fold), and *BRCA-2* (1.60-fold) gene expressions in the Cur and Doxo combination treatment (Figure 12, Figure 13), compared to the untreated MDA-MB-231 cells. These outcomes conclude the *P53*-mediated S-phase arrest triggered the apoptosis [21,22] mechanism in the combination dose-treated MDA-MB-231 cells. The overall apoptosis mechanism [23] is schematically presented in Figure 14 (previously reported in the preprint [24]).

Study limitations

Overall, this preclinical study aims to provide a promising direction for developing a novel strategy to inhibit tumor cell growth and establish Cur as a potential adjunct to frontline breast cancer therapeutics, specifically Adriamycin (Doxo). However, using embryonic kidney cells as the control could be a limitation of the study. Therefore, further in vitro investigations may be required to validate the findings, and additional clinical trials are essential for its validation as a commercial therapy.

## Conclusions

The present in vitro study showed the potential growth-inhibitory activities of Cur against human breast cancer cells (MDA-MB-231). Its synergy at broad ranges was found to be consistent with the combination treatment with Doxo at an optimal specific dose (33.12 μM Cur + 0.33 μM Doxo) as a chemo-sensitizing agent for cancer therapy. The cytotoxicity of the combination dose was cross-verified on normal kidney epithelial cells (HEK293), where the minimal toxicity proved the chemo-protectiveness of Cur against Doxo. The underlying mechanisms causing the anticancer activities of Cur with its pleiotropic effects were also revealed in the combination treatment. The elevated oxidative stress (ROS) was found to enhance the apoptosis of cancer cells enormously after being treated with the Cur and Doxo combination treatment. Meanwhile, the chromatin condensation and double-stranded DNA break further activated the *P53*-mediated S-phase cell cycle arrest in the MDA-MB-231 cell lines. Also, the upregulated expression of the *ATM* gene in the combination dose-treated MDA-MB-231 cells emphasized the *CHEK2*-mediated signal transduction, which caused the G_1_/S checkpoint activation by cyclin-A/E. The slightly elevated *BRCA-1* expressions induced the further activity of *BRCA-2* at the S/G_2_ and G_2_/M checkpoints to prevent further cell division in MDA-MB-231 cells.
